# Unlocking the Potential for Implementation of Equitable, Digitally Enabled Citizen Science: Multidisciplinary Digital Health Perspective

**DOI:** 10.2196/50491

**Published:** 2024-12-10

**Authors:** Lucio Naccarella, Jonathan Charles Rawstorn, Jaimon Kelly, Eleanor Quested, Stuart Jenkinson, Dominika Kwasnicka

**Affiliations:** 1 Melbourne School of Population and Global Health The University of Melbourne Melbourne Australia; 2 Institute for Physical Activity and Nutrition School of Exercise and Nutrition Sciences Deakin University Geelong Australia; 3 Centre for Online Health The University of Queensland Brisbane Australia; 4 Curtin School of Population Health Faculty of Health Sciences Curtin University Perth Australia; 5 Systemic Policy and Strategy Team Carers Western Australia Perth Australia

**Keywords:** citizen science, digital health, equity, implementation science, community, research, health inequality, health equity, health integration, mental well-being, well-being

## Abstract

Citizen science is a community-based participatory research approach with an emphasis on addressing health disparities that is increasingly advocated by the community, researchers, and research funders. Digitally enabled methods can extend the potential of citizen science by enabling citizens to engage in real-time research processes, such as data collection, information sharing, interpreting, acting on data, and informing decision-making. However, the power of any citizen science lies in promoting health equity by providing equal opportunity for citizen engagement. Without appropriate attention to recognize and address equity, digital enablement of citizen science may exacerbate rather than ameliorate health inequalities. In this Viewpoint, we draw on our digital health research experience and perspectives to outline the practice of citizen science in the context of digital health—how it is operationalized, key advocated principles, and challenges. We also discuss citizen science in relation to health equity and implementation science, including emphasizing the importance of integrating health equity principles and frameworks, health equity implementation determinants, and digital determinants of health. We demonstrate how equity could be achieved by providing a working example in the context of a digitally enabled approach to improving social, physical, and mental well-being among people with disability and caregivers.

## Introduction

Citizen science is a community-based participatory research approach that specifically involves the participation of community members, who may not have formal scientific training, in the scientific process—often by collecting, analyzing, and interpreting data. Participatory research methodologies like citizen science are increasingly advocated by consumers, the community, researchers, and research funders [1]. While guiding principles exist [2,3] and many virtues are claimed [4], implementation into practice remains a challenge for most researchers, practitioners, and policy makers [5,6]. Digital technologies have enabled the global growth of citizen science and led to the development of the field which here we coined as digitally enabled citizen science*,* where digital technologies support citizen scientists in engaging in real-time research processes.

The successful integration of citizen science, digital health equity, and health equity implementation into digital health care could be achieved by using existing principles, frameworks, and known health equity implementation determinants and digital determinants of health. In this interdisciplinary viewpoint, we outline key principles and challenges of citizen science. We highlight the intersection of citizen science and digital health, and emphasize the importance of health equity in implementation. We present a digitally enabled citizen science project (ConnectUp) as a working example of how relevant principles, frameworks, and determinants can be integrated (Textbox 1).

**Case study: ConnectUp.**

ConnectUp is a prototype web-based platform that connects caregivers and people with disability with geographically proximal peers to engage in physical activities. Users create profiles describing personal characteristics (age, physical activities they enjoy engaging in, how far they are willing to travel to meet with someone who they matched with), and ConnectUp matches people with similar profiles to facilitate connections and engagement.

The platform was co-designed by a range of stakeholders including a state-level leading not-for-profit organization supporting caregivers (Carers WA), a digital health agency (Eduka Pty Ltd), and people with disability and their caregivers. A total of 17 participants took part in a co-design process, led by a person with disability, which included 4 web-based or 2 face-to-face workshops. Participants represented a broad range of ages (range 20-73 years), cultural backgrounds (Australian, Cambodian, Singaporean, Romanian, and English), and disabilities (intellectual, physical, and mental health conditions).

The co-designed prototype is currently being extended to support digitally enabled citizen science and enhance the user experience. ConnectUp users will be able to upload new physical activity opportunities into the platform (including defining characteristics such as location, activity type, and accessibility considerations), update characteristics of existing opportunities (eg, changing accessibility, new characteristics relevant to different types of disability), and review existing opportunities to provide lived-experience perspectives that can help other ConnectUp users to determine suitability for their needs. ConnectUp will empower citizens to create, curate, and disseminate information that can strengthen physical, social, and psychological well-being among people with disability and caregivers, as well as generate large-scale evidence about the accessibility and inclusivity of community amenities to support advocacy for policy and business reform.

Citizen science is typically operationalized on a spectrum with varying levels of citizen involvement. Multiple taxonomies reflect this variation [7-9]. Table 1 provides 2 examples.

Higher levels of involvement suggest greater commitments to creating equal opportunity for citizens to engage in the research process, key to promoting health equity. In ConnectUp, we support higher levels of citizen involvement, empowering citizens to be involved at all research stages, including them in Steering Committee and Advisory Committee, and using data to inform and activate community action. While citizen science is increasingly advocated to address long-standing health inequity, the issue of “power dynamics” between researchers and citizens is debated by the community, researchers, and research funders, especially in the context of the COVID-19 pandemic [9] For example, lower involvement may widen power imbalances and potentially exploit vulnerable community groups because it provides citizens with less control and voice than higher involvement.

**Table 1:** Examples of citizen science taxonomies.

Level of involvement	Example taxonomy 1 (English et al [7])	Example taxonomy 2 (King et al [8] and Tan et al [9])
Low	Crowdsourcing: involves active or passive citizen participation in data collection	For the people: citizens are involved in informing and contributing to research 1-way via meetings
Low	Limited participatory research: involves citizens in problem definition and data collection	With the people: citizens are involved in consulting and collaborating in research processes via consultations, interviews, or surveys
High	Extreme citizen science: involves citizens in analysis and interpretation, study dissemination, and public health action	By the people: citizens are empowered to be involved in all research processes, via advisory committees or community forums and can include applying data to inform and activate community action

Citizen science has been applied and adapted within diverse research contexts, population groups, and content areas, and guiding principles have been developed by regulatory bodies to promote consistent and high-quality practices. For example, the Australian and European Citizen Science Associations both recommend 10 community citizen science engagement principles to ensure research quality, equity, inclusion, and governance [10,11] (Table 2). Table 2 demonstrates how these principles have been actioned in the ConnectUp case study.

**Table 2:** Citizen science engagement principles [10,11], exemplified with ConnectUp.

Engagement principles	ConnectUp: case study principles in action
1. Project actively involves citizens and generates new knowledge or understanding.	People with disability and caregivers co-defined the problem (lack of physical activity and opportunities to be active and socialize) and solution (web-based platform creating connections with like-minded people and physical activity opportunities in nearby locations).
2. Project has a genuine scientific outcome.	The platform that was proposed by users will expand the initial scope of the project to facilitate social connection. It will include elements of data collection (listing and reviewing places to be active), data summaries and feedback (rating of facilities and activities on offer), and data will be collated, analyzed, and co-summarized with and by consumers (people using the platform). The project will include a robust evaluation of effects on meaningful health/social outcomes, the evaluation will be developed and refined with all stakeholders.
3. Project provides benefits to both science and society.	With the active participation of consumers in gathering data, researchers will be able to better understand several issues including barriers and facilitators to access physical activity opportunities. The benefits for society will include service provision—the users will be able to access and use up-to-date crowdsourced information that is relevant to them and in the area that they live in. They will be able to actively participate in the research process if required.
4. Citizen scientists may participate in various stages of the scientific process.	Stages that citizen scientists participate in include study and platform design (partially completed); data collection, data indexing and categorizing, data processing, analyzing, and summarizing (together with the researchers). Followed by involvement in outcomes interpretation, dissemination and local advocacy, participation in publications and presentations as well as informing local policy.
5. Citizen scientists receive feedback from the project team.	The academic researchers seek to identify citizen scientists’ capacity development needs and provide appropriate feedback, training, and resources to support development.
6. Project has limitations and biases that should be considered and controlled for.	Not all citizen scientists may see themselves as equal participants in the research process. Through extensive training, conversations, responsibility sharing, and project goal setting, the researchers will continue to strive to ensure equity of involvement between citizen scientists and researchers.
7. Project data and meta-data are publicly available and results are published in accessible format.	Project data and meta-data will be publicly available and results will be published on the ConnectUp website, the summary of research results will be published in accessible reports. Data will also be available on the Open Science Framework to increase data discoverability in the scientific community.
8. Citizen scientists are suitably acknowledged by project team.	Citizen scientists will continue to colead the academic researchers, for instance, coauthor SJ received training in qualitative research methods and led co-design workshops/focus groups. Lead citizen scientists will be named on the study website and will coauthor scientific publications (eg, SJ and others).
9. Project offers benefits and outcomes are considered in project evaluation.	Citizen scientists will continue to be involved in defining key project benefits and setting outcomes and they will take an active role in project evaluation during the full duration of the project.
10. Project takes into consideration relevant legal and ethical issues.	Legal and ethical aspects of the project are considered by the full team throughout the research project by discussing and providing training in legal and ethical principles of citizen science–led research and codeveloping ethical standards to follow the citizen science best practices.

Several insights from the community members pointed to the need for the proposed work:

We've done some research on Google for something and then you really excited or keen and then you look at it and go okay, that's not that program, it's not running or it's finished. And we do believe it would be a full-time job, or potentially a team of people updating the information but the importance of that to us as carers, I think is invaluable. It would be worth having because we are so time poor we want something that is a one stop shop.... You know, there's a lot of “come and try” days for people. But how many people know about it? Unless you're on the right mailing list, you don't know about them or what they offer. I think it's bringing all those together in the one umbrella, and everybody promotes the one thing. At the moment, I get all my resources from different newsletters, and I think you need something that's geographically located.

caregiver, ConnectUp user co-design workshop 1, hosted by Carers WA

People with disability echoed this, raising similar issues:

I think if you start this database. You will have people then saying, hey, how about us, did you know about this? You can have others that can then put forward ideas. I think that is really important, because, you know, we all have different things that we are knowledgeable about, and it's just bringing it together.

person with disability, ConnectUp user co-design workshop 3, hosted via web

While the promises, virtues, and benefits of citizen science are increasingly being espoused [12,13], debates about operational and conceptual challenges in practice are also increasing (Textbox 2).

**Citizen science operational and conceptual challenges.**

**Operational challenges**

- Ensuring equal representation of citizens in all research processes.
- Balancing scientific and social value of citizen-generated knowledge and citizen capabilities.
- Balancing connections between citizens and researchers and research quality.
- Managing the complex link between citizen science and participatory policy development.

**Conceptual challenges**

- Diverse motivations of citizens for participating in research.
- Dynamic public trust and distrust in scientific knowledge and endeavors.
- Issues of power, exploitation, and commitment to engagement in citizen science projects/programs.

Challenges related to citizen representation and power signal that health equity and implementation (ie, the use of strategies to adopt and integrate evidence-informed interventions and to change practice patterns) requires focused consideration, reflection, and action to ensure inequities are not perpetuated or increased.

## Citizen Science in Digital Health

Digital health refers to the development and use of digital technologies to improve health, for example, virtual health, mobile health apps, wearable devices, the internet, and artificial intelligence tools that enable the storage, exchange, advanced analysis, and visualization of data [14]. Digital technologies have contributed to the rise of citizen science and led to the field which we coined here digitally enabled citizen science*,* where digital methods are used to engage citizens in real-time research processes [15]. However, the integration of “digital health” with “Citizen Science” is multi-faceted and complex. Digital health tools could facilitate equitable citizen participation in health intervention design, development, adoption, implementation, evaluation, and sustainability. This is achieved by providing a voice to citizens who experience health inequities and having the reach and power to source big data to inform health policy decision-making. However, digital health can also contribute to health inequities when designed, developed, implemented, and evaluated without adequate consideration of the digital determinants of health, such as digital health literacy [16]. The rapid digital transformation of health care may contribute to increased inequity as uneven adoption of health interventions can widen existing gaps between advantaged and disadvantaged populations [17].

## Equitable Implementation of Digitally Enabled Citizen Science Initiatives: Guiding Frameworks

Health equity means that no one is denied access to optimal health and well-being because they are economically or socially disadvantaged [18]. Health interventions can perpetuate health disparities when implementation leads to inequitable inputs (eg, funding), outputs (eg, uptake and quality), and outcomes (eg, access to care). Race, ethnicity, sexual orientation, gender identity, socioeconomic status, functional limitations, and other characteristics can contribute to disparities in the implementation of health interventions. As implementation science [19] progressed, there was increased use of implementation determinant frameworks to understand why these disparities occur [20]. For instance, the health equity implementation framework [21] recommends the integration of three health equity domains into existing implementation science frameworks, namely: (1) health care intervention recipient cultural factors (eg, socioeconomic status, race or ethnicity, and language); (2) interaction between clinicians and patients (eg, interactions can predict satisfaction, perceived trust, and health outcomes); and (3) societal context (eg, economic, demographic, or geographical factors).

These health equity domains can guide the use of existing implementation science frameworks and efforts to identify and understand barriers to equitable implementation of Digitally enabled citizen science initiatives. The promise and power of citizen science to address health inequities lie in the opportunity for broader, more equitable citizen participation and engagement in research processes—to identify, systematically collect, analyze, and use data that are meaningful and relevant to citizens, researchers, and policymakers. The known disparities in access to digital technology and opportunities within and between individuals, communities, and nations [22], reinforce the necessity of equitable digitally enabled citizen science, if the use of the paradigm is to result in large-scale health benefits.

A recent review [11] that aimed to summarize existing efforts to use citizen science to address health equity recommended expanding the focus on topics important for health equity (eg, equitable access). The suggestions included increasing the diversity of people serving as citizen scientists and their involvement and integration in research process phases (eg, applying the data to inform and activate community action), continuing to leverage emerging technologies that enable citizen scientists to collect data relevant to health equity, and strengthening the rigor of methods to evaluate impacts on health equity. Application of these recommendations has the potential to unlock the equitable implementation of citizen science projects, in particular where digital health tools are used.

## Citizen Science and Digital Health Equity: Principles, Frameworks, and Determinants

Digital health equity refers to having an equal opportunity for individuals to benefit from the knowledge and practices related to the development and use of digital technologies to improve health [22]. Digital health technologies interact with social, cultural, and economic realities and with social determinants of health to indirectly contribute to health equity or inequity. Given that digital health tools are being used to engage citizens in real-time research processes [8], digital health equity principles, frameworks, and determinants need consideration to facilitate equitable citizen participation. While a universal set of digital health equity principles does not exist, principles relevant to digital health interventions have been suggested [23]. For example, equity principles have been derived from a digital equity assessment tool [23]. The digital health equity framework (DHEF) builds upon the health equity measurement framework [24] designed to measure the effects of social determinants of health to support improved statistical modeling and the measurement of health equity. Textbox 3 synthesizes equity principles relevant to digital health and illustrates how they have been actioned in the ConnectUp case study.

We advocate that these principles should also guide the design, development, implementation, and evaluation of digitally enabled citizen science initiatives. To date, several DHEFs [25-27] have been developed as calls to action that seek to guide the planning, implementation, and evaluation of equity-informed digital health interventions. The DHEFs are informed by and build upon integrative literature reviews and syntheses of existing health equity frameworks. These frameworks also signal the importance of digital determinants of health, the unique elements of people’s experiences with the digital health environment (eg, digital health literacy, access to digital resources, and infrastructure) [17].

The DHEF [27] reinforces how the digital determinants of health interact with a person’s current health state and needs, and other intermediate health factors such as psychosocial stressors, pre-existing health conditions, health-related beliefs and behaviors, and the environment. For example, access to digital health resources and digital health literacy interact with the degree and kind of psychosocial stress a person is currently experiencing; job loss or poverty, level of education, and previous exposure to digital media can all impact access. The framework also highlights the importance of approaching digital health technologies from an ecological perspective, considering the ways an individual’s use of technologies extends out into (and is shaped by) their social, cultural, and economic position.

The framework for digital health equity [25] also highlights the ecological perspective to encourage multiple approaches to digital health by identifying individual-level, interpersonal-level, community-level, and societal-level determinants. For example, interventions targeting “upstream” determinants (eg, digital infrastructure) at the community and societal levels have the potential for the greatest impact on more populations. Collectively DHEFs can provide a comprehensive guide to planning, implementing, and evaluating digitally enabled citizen science initiatives to maximize equity.

**Digital health equity principles.**

**Digital health equity principles**

The digital health initiative should

- facilitate equal community participation;
- improve the status of populations who are disadvantaged;
- narrow the health divide between population groups;
- reduce social inequalities throughout the whole population;
- not be driven by or exploit users for profit;
- be provided according to need, not ability to pay;
- tackle fundamental social determinants of health;
- provide a voice to the voiceless;
- facilitate equal access to services;
- evaluate the impacts by sex, gender, race/ethnicity, geographic and socioeconomic communities.

**ConnectUp reflections on principles in action**

ConnectUp is designed to

- create a community that is inviting to all. Equity challenges include digital health literacy, access to internet-enabled devices, and the impacts of some on user interactions (eg, blindness, neurological impairments);
- improve social connection and physical activity;
- reduce excess health burden and inequity associated with disability and caring;
- reduce social inequalities throughout the target population, making the platform user-friendly and accessible, and promoting it at the population level;
- protect users by not using data for advertising or sale to third parties;
- be freely available;
- improve social inclusion and nondiscrimination, and increase access to affordable, quality health services;
- enable marginalized people to build a community that exchanges knowledge and experiences of social connection and physical activity;
- identify and promote dissemination of social and physical activity opportunities that are suitable for people with disability;
- include a comprehensive process evaluation, designed jointly with people with disability and caregivers, to evaluate the impacts separately for differing sex, gender, race/ethnicity, and geographic and socioeconomic communities.

## Conclusion

Digitally enabled citizen science is increasingly being advocated by the community, researchers, and research funders, particularly to address health disparities, and to unlock its equitable implementation potential. We acknowledge that challenges exist, particularly when an action requires collaboration between multiple fields that may have differing understandings and use of key terms and concepts (ie, citizen science, equity, and digital health). Our goal is to discuss citizen science and to create the opportunity by encouraging this coming together of different paradigms to encourage collaboration, and ultimately improve and widen the equitable application of digitally enabled citizen science. We advocate that future Digitally enabled citizen science requires the integration of digital health equity and health equity implementation. That is, to ensure initiatives are designed, developed, implemented, evaluated, and sustained using (1) principles (existing citizen science engagement principles and digital health equity principles provide a critical platform), (2) frameworks (existing health equity implementation and DHEFs provide a comprehensive guide for planning, implementation, and evaluation activities), and (3) determinants (the known health equity implementation determinants and digital determinants of health will optimize the identification of strategies to improve equitable implementation of digitally enabled citizen science initiatives).

## Abbreviations

DHEF: digital health equity framework
